# Carpet Beating

**Published:** 1884-06

**Authors:** 


### CARPET BEATING.

WE have no wish to decry this periodic purification, but merely to point out rules for its better conduct and efficiency. The usual process in households which cannot afford to have it carried out by special agency is first to take up the carpets and sweep the walls and ceilings, and then to wash the floors. While the latter are drying, work must be found for the idle hands, and they cannot be better employed than in beating the carpets in the court-yard or back garden, a work attended with a horrid din and clouds of dust. It will be unavailing, we know, to complain of the noise—no appeal in this direction will gain a moment's sympathy; but we hope more attention will be paid to the other nuisance. When we reflect on the nature of the dust thus raised, we are surprised that sane persons allow to be thus stirred up under their noses all the nauseous accumulations of dining-room, bed-room, and stair carpets, to say nothing of door mats, etc., into a fine dust, and which, thus dispersed, finds its way again into our houses in a form most readily accessible to our respiratory organs. Indeed, it is fortunate if the dust thus roused is only nauseous and not effective, since the desquamated cuticle of scarlet fever, the scabs of smallpox, the dried sputa of consumptive or whooping-cough patients, living parasites, and hairs from mangy cats and dogs may thus invade our rooms. Carpet-beating, we are aware, is forbidden in public thoroughfares; but it should, in any form, be prohibited within a reasonable distance of dwelling-houses; and for those who cannot afford to pay the small sum required to have their carpets and mats properly cleaned, the authorities should set aside some open space, to which on stated days and at certain hours persons might bring their carpets and have them beaten, without causing annoyance or danger to themselves or neighbors.—*London Lancet.*

